# Establishment of an Academic Tissue Microarray Platform as a Tool for Soft Tissue Sarcoma Research

**DOI:** 10.1155/2021/6675260

**Published:** 2021-03-15

**Authors:** Che-Jui Lee, Agnieszka Wozniak, Thomas Van Cann, Iris Timmermans, Jasmien Wellens, Ulla Vanleeuw, Inge H. Briaire-de Bruijn, Christian Britschgi, Judith V. M. G. Bovée, Inti Zlobec, Raf Sciot, Patrick Schöffski

**Affiliations:** ^1^Laboratory of Experimental Oncology, Department of Oncology, KU Leuven, Leuven, Belgium; ^2^Department of General Medical Oncology, University Hospitals Leuven, Leuven, Belgium; ^3^Department of Pathology, Leiden University Medical Center, Leiden, Netherlands; ^4^Department of Medical Oncology and Hematology, University Hospital Zürich, Zürich, Switzerland; ^5^Translational Research Unit, Institute of Pathology, University of Bern, Bern, Switzerland; ^6^Department of Pathology, University Hospitals Leuven, KU Leuven, Leuven, Belgium

## Abstract

Soft tissue sarcoma (STS) is a heterogeneous family of rare mesenchymal tumors, characterized by histopathological and molecular diversity. Tissue microarray (TMA) is a tool that allows performing research in orphan diseases in a more efficient and cost-effective way. TMAs are paraffin blocks consisting of multiple small representative tissue cores from biological samples, for example, from multiple donors, diverse sites of disease, or multiple different diseases. In 2015, we began constructing TMAs using archival tumor material from STS patients. Specimens were well annotated in terms of histopathological diagnosis, treatment, and clinical follow-up of the tissue donors. Each TMA block contains duplicate or triplicate 1.0–1.5 mm tissue cores from representative tumor areas selected by sarcoma pathologists. The construction of TMAs was performed with TMA Grand Master (3DHistech). So far, we have established disease-specific TMAs from 7 STS subtypes: gastrointestinal stromal tumor (72 cases included in the array), alveolar soft part sarcoma (*n* = 12 + 47), clear cell sarcoma (*n* = 22 + 32), leiomyosarcoma (*n* = 55), liposarcoma (*n* = 42), inflammatory myofibroblastic tumor (*n* = 12 + 21), and alveolar rhabdomyosarcoma (*n* = 24). We also constructed a multisarcoma TMA covering a representative number of important histopathological subtypes on arrays for screening purposes, namely, angiosarcoma, dedifferentiated liposarcoma, pleomorphic liposarcoma, and myxoid liposarcoma, leiomyosarcoma, malignant peripheral nerve sheath tumor, myxofibrosarcoma, rhabdomyosarcoma, synovial sarcoma, and undifferentiated pleomorphic sarcoma, with 7–11 individual cases per subtype. We are currently expanding the list of TMAs with additional sarcoma entities, considering the heterogeneity of this family of tumors. Our extensive STS TMA platform is suitable for rapid and cost-effective morphological, immunohistochemical, and molecular characterization of the tumor as well as for the identification of potential novel diagnostic markers and drug targets. It is readily available for collaborative projects with research partners.

## 1. Introduction

Soft tissue sarcoma (STS) is a heterogeneous group of rare malignant tumors derived from mesenchymal progenitor cells, which are defined by various morphological, histopathological, and genetic characteristics [1, 2]. Tissue analysis, such as histology and immunohistochemistry (IHC), is essential for the clinical classification of sarcomas, treatment planning, and prognostic assessment. In the past few years, scientific progress in the complex field of STS has mainly been driven by the identification and implementation of novel prognostic and predictive markers, as illustrated by the success of kinase inhibitors treatments for gastrointestinal stromal tumors (GIST) [3], the use of epigenetic modifiers for epithelioid sarcomas with integrase interactor 1 loss [4], or the agnostic use of neurotrophic receptor tyrosine kinase (NTRK) inhibitors in sarcomas with specific gene fusions [5]. The approval of such drugs shows that the identification of novel biomarkers with diagnostic, prognostic, or predictive value, and a better understanding of the biology of an individual sarcoma subtype can rapidly translate into therapeutic relevance and improve the clinical outcome of patients. STS represents a family of at least 100 individual subtypes [1, 6], and all sarcomas together account for only 1% of all adult solid tumors [6]. Tissue collections are important to achieve further scientific progress and to gain a deeper insight into the biology of some sarcoma entities and the potential relevance of biomarkers. The use of archival sarcoma material for histopathology, immunohistochemical, and genetic studies is time-consuming, expensive, and labor-intensive, especially if a large number of cases are required for explorative studies.

Tissue microarray (TMA) can overcome some limitations of using conventional archival sarcoma tissue for research. TMA is a paraffin block consisting of many small representative tissue cores from patient samples in alignment of an array. This technology was first described by Kononen et al. in 1998 [7] but the concept can be traced back to 1986 when Battifora designed a “sausage block” by wrapping 1 mm thick rods of different specimens and embedding them afterwards in a paraffin block [8]. TMA can combine tissue from multiple donors with the same sarcoma subtype, multiple types of sarcoma from various donors, or longitudinal samples from the same donor. With modern TMAs, a large number of archival samples can be analyzed in parallel using tissue-based applications such as IHC, fluorescent *in situ* hybridization (FISH), or (multiplex) immunofluorescence. Such TMAs have potential advantages over conventional, single sample-based tissue analysis in terms of efficiency and cost-effectiveness of the work, especially when tissue is analyzed for research purposes. In 2015, the Laboratory of Experimental Oncology, KU Leuven, Leuven (Belgium) started establishing STS TMAs using left-over archival sarcoma tissue from patients treated at the University Hospitals in Leuven. Our primary aim was to create a ready-to-use TMA platform for sarcoma research. This article provides an overview on available TMAs and describes how they were constructed, examples of applications for translational research, and where we see potential advantages and limitations of this approach.

## 2. Materials and Methods

### 2.1. Collection of Archival Tumor Tissue Blocks and Clinical Data

For the construction of TMAs, formalin-fixed (10% neutral buffered formalin for 24 hours), paraffin-embedded (FFPE) tumor blocks were collected from STS patients diagnosed at the University Hospitals Leuven (UZ Leuven), with left-over tissue archived in the Department of Pathology, UZ Leuven. We identified and retrieved relevant tumor blocks through the retrospective review of our sarcoma-specific clinical and research database (LECTOR), established and maintained by the Department of General Medical Oncology, UZ Leuven. The initial focus on certain sarcoma subtypes was primarily done with the purpose to match and support ongoing translational and clinical research projects in our group; in a second step, we started establishing TMAs from a variety of sarcomas to facilitate future translational research in the broader STS family of diseases. To enrich the collection with a sufficient number of cases from some ultrarare STS subtypes, a limited number of additional tumor blocks were collected from selected collaborating institutions: University Hospital Zürich (USZ), Zürich (Switzerland) and Leiden University Medical Center (LUMC), Leiden (The Netherlands). Furthermore, we utilized tissue blocks that were collected in the frame of the European Organisation of Research and Treatment of Cancer (EORTC) phase 2 trial 90101 “CREATE” [9–11], where left-over FFPE blocks from patients with clear cell sarcoma (CCSA), alveolar soft part sarcoma (ASPS), and inflammatory myofibroblastic tumor (IMFT) were centrally stored at a commercial biorepository, BioRep, Milan (Italy), for research purposes.

Apart from focusing on specific sarcoma subtypes, we applied the following criteria for selecting suitable donor tissue: available blocks with a sufficient amount of tumor tissue, without dehydration (to avoid brittle blocks) and depth of block at least 3 mm (to make sure a sufficient number of sections can be cut). All diagnoses were established or reviewed by sarcoma pathologists at UZ Leuven (RS) or LUMC (JVMGB) and confirmed by the presence of characteristic, immunohistological, or molecular markers if applicable, depending on the tumor type. The corresponding clinical data including pathological diagnosis, treatment, and clinical follow-up were extracted from LECTOR (UZ Leuven) and the CREATE-related trial database at EORTC, Brussels (Belgium). The collection and the analysis of pseudonymized clinical data and use of archival FFPE tumor samples were approved by the Medical Ethics Committee, UZ Leuven (reference numbers S51495, S59181). The collection of ASPS and IMFT cases from the archive of the Department of Pathology, Leiden University Medical Center, was approved with a waiver of consent by LUMC's Medical Ethics Committee (B17.030).

### 2.2. Construction of TMA

Slides were cut from retrieved archival FFPE blocks, stained with hematoxylin and eosin (H&E), and assessed microscopically to evaluate the quality of tumor material. Selected high-quality blocks were sent to the Translational Research Unit (TRU), Institute of Pathology, University of Bern, Bern (Switzerland) or LUMC. The central facility made further H&E-stained sections which were digitally scanned for unbiased annotations of areas of interest. Cores of preselected diameter were made from these areas using CaseViewer 2.3 (3DHistech, Budapest, Hungary) (CJL) and reviewed by reference sarcoma pathologists (RS and JVMGB). The construction of TMAs was performed with the fully automated machine TMA Grand Master from 3DHistech, Budapest (Hungary). The TMA control software (TMA Grand Master package) was used to align images with corresponding donor blocks and to locate the annotated area. Two to three cores of 1.0 or 1.5 mm in diameter were automatically punched out from a donor block by the TMA machine according to the digital annotations. The cores were then relocated to a recipient block in a precise alignment. The annotated information and the corresponding location of cores were automatically archived and stored in a specific file. Subsequently, 4 *μ*m sections were cut from the constructed TMA blocks, H&E-stained, and scanned for quality control purposes.

### 2.3. Immunohistochemistry

For a typical immunohistochemical study, TMA sections were pretreated with Ultra Clear (VWR, Pennsylvania, US) and 100% ethanol for deparaffinization and dehydration, and were incubated in a solution of methanol (Acros Organics, New Jersey, US) with hydrogen peroxidase (Merck, New Jersey, US) for blocking endogenous peroxidase. Antigen retrieval was achieved by sections incubated in 10 mM citrate buffer (pH 6.0) in a preheated water bath at 95°C for 30 minutes, followed by the incubation of protein block serum-free (DAKO A/S X0909, Glostrup, Denmark). For the purpose of comparison between whole tumor tissue section and TMA, immunostaining was performed with phospho-p44/42 mitogen-activated protein kinase antibody (pMAPK, Cell Signaling Technology #4370, Massachusetts, US) and phospho-AKT antibody (pAKT, Cell Signaling Technology #9271) at 4°C overnight. For target screening in multiple STS subtypes, immunostaining was performed with human platelet-derived growth factor receptor beta (PDGFR-B) antibody (R&D AF385, Minneapolis, US) at room temperature for one hour. Visualization was done by 3,3′-Diaminobenzidine Substrate Chromogen System (DAKO A/S K34681), following instructions from the manufacturer. After counterstaining with hematoxylin (VWR), slides were dehydrated in series of 100% ethanol solution and mounted. The stained slides were automatically scanned and evaluated blindly and independently by two investigators using Olympus BX43 microscopy and cellSens software (Olympus, Tokyo, Japan). Scoring was done according to scoring intensity: 0 (negative), 1 (weakly positive), 2 (moderately positive), and 3 (strongly positive). For cases with more than one available core on the TMA, a mathematical mean was recorded as the final result.

### 2.4. Multiple Iteractive Labeling by Antibody Neodeposition

We also explored the use of TMAs for multiplex immunoassay and applied the Multiple Iteractive Labeling by Antibody Neodeposition (MILAN) technique, an assay involving repeated cycles of indirect immunofluorescence, image acquisition, and antibody removal [12, 13]. We optimized the procedure for TMA sections and fluorescence microscopy. Deparaffinization and dehydration were performed as described above, followed by antigen retrieval with Tris-EDTA (pH = 9). Multicolor immunofluorescent staining was achieved using an antibody cocktail made by 1 : 50 dilution of primary antibodies, which were selected from different host species or different subclass of immunoglobulin G (IgG) (Supplementary Table S1). TMA sections were incubated with cocktail antibody solution at 4°C overnight. Corresponding fluorophore-conjugated secondary antibodies (1 : 200 dilution) were sequentially administrated to the TMAs at room temperature for one hour, followed by fluorescence counterstaining with 4′,6-diamidino-2-phenylindole (DAPI, Thermo Fisher Scientific, Massachusetts, US) and mounting with dissolvable medium. After image acquisition, the coverslip was removed by incubation in washing solution, and antibodies were removed with preheated stripping buffer (2-mercaptoethanol and sodium dodecyl sulphate, Sigma-Aldrich, Missouri, US) at 56°C for 30 minutes with horizontal shaking. After this, the next cycle could be applied with different combinations of primary and fluorophore-conjugated antibodies. The number and sequence of cycles were determined by the potential expression level of interested targets and to minimize the possible impact of tissue loss during the experiment (Supplementary Table S1). In the current manuscript, we present the combined use of TMAs and MILAN as an illustration of the potential use of this technique for characterization of the tumor microenvironment in ASPS, one of the few sarcoma subtypes tending to respond to immune checkpoint modulation in the clinic. In this experiment, we performed multiplex staining for immune checkpoints, markers for tumor-infiltrating lymphocytes, and tumor-specific molecules (Supplementary Table S1). Immunofluorescence staining was scored blindly in the previously defined order by the investigator (CJL) and was assessed as a categorical variable based on the percentage of cells expressing targeted molecules. The targeted molecules and corresponding evaluation criteria were the following: cell membrane expression on multiplex immunofluorescence of the markers programmed cell death protein 1, programmed cell death protein ligand 1, cytotoxic T lymphocyte-associated protein 4, CD3, CD4, CD8, CD14, CD56, CD68, and major histocompatibility complex class I/II were considered as positive. For transcription factor E3 (TFE3, ASPS specific molecule), nuclear expression was considered as positive [14]. Both cytoplasmic and nuclear expressions of forkhead box protein P3 were defined as positive [15]. The proportion of cells was scored as 0 (no cells stained), 1 (1–10% of stained cells), 2 (10–30% of stained cells), or 3 (>30% of stained cells).

## 3. Results

### 3.1. Tissue Donor Characteristics and Tumor Blocks

Between April 2015 and March 2020, 459 selected STS FFPE blocks, originating from patients who had undergone surgery or biopsy procedures, were retrieved from archives of UZ Leuven and collaborating institutions (USZ provided 4 cases of CCSA; LUMC donated 4 cases of ASPS and 5 cases of IMFT). These samples originated from a total of 328 individual patients, with a male-to-female ratio of 0.97 and a median age of the patients at sarcoma diagnosis of 58 years (range 0–95). The majority of donor samples were collected from a primary tumor (41%) or a metastatic lesion (40%), followed by local recurrence (16%). The selected samples originated mainly from abdominal or chest sites (58%), extremities (22%), trunk (7%), or head and neck (6%). The most common subtypes of STS included were leiomyosarcoma (LMS, 31%), GIST (17%), CCSA (8%), alveolar rhabdomyosarcoma (ARMS, 7%), dedifferentiated liposarcoma (DDLPS, 6%), myxoid/round cell liposarcoma (MLPS, 6%), pleomorphic liposarcoma (PLPS, 4%), well-differentiated liposarcoma (WDLPS, 4%), ASPS (3%), IMFT (3%), angiosarcoma (2%), malignant peripheral nerve sheath tumor (MPNST, 2%), myxofibrosarcoma (2%), rhabdomyosarcoma (RMS, 2%), synovial sarcoma (SynSa, 2%), and undifferentiated pleomorphic sarcoma (UPS, 2%). The characteristics of each tumor type are summarized in Supplementary Table S2.

In addition, 102 available archival tissue samples from 100 individual donors with ASPS, CCSA, or IMFT, who participated in EORTC trial 90101, were used for TMA construction, with a male-to-female ratio of 1.29 and a median age at diagnosis of the patients with these rare sarcomas of 33 years (range 1–77). The majority of these samples were collected from a primary tumor (71%) or a metastatic lesion (27%). These orphan sarcomas are known to be potentially driven by specific molecular alterations, and the presence of the according genetic hallmark was therefore determined by FISH and/or IHC as part of the original study protocol [16–18]. Specific translocations were confirmed by the presence of a rearrangement of *TFE3* in ASPS (89% of cases) and rearrangement of Ewing sarcoma breakpoint region 1 (*EWSR1*) in CCSA (88%). Anaplastic lymphoma kinase (*ALK*) rearrangement and/or immunopositivity was analyzed in IMFT (62%) (Supplementary Table S3).

### 3.2. Overview of Currently Available TMAs

In an ongoing effort, we have created a current total of 10 STS subtype-specific TMAs, including two TMAs each from ASPS (12 and 47 cases per array), CCSA (22 and 32), and IMFT (12 and 21), as well as one TMA each from GIST (72), LMS (55), LPS (43), and ARMS (21). For screening purposes, we have also constructed a multisarcoma TMA covering 10 characteristic, clinically relevant subtypes of STS, combining 7–11 individual cases of angiosarcoma, DDLPS, PLPS, MLPS, LMS, MPNST, myxofibrosarcoma, RMS, SynSa, and UPS on one array. A detailed overview of the current status of our TMA platform with basic description of the individual array features is presented in Table 1. Additional TMAs from solitary fibrous tumor (SFT), angiosarcoma, and SynSa are currently under construction or in preparation, to match ongoing translational research in our group. Some TMAs (e.g., UZL_TMA_CCSA) were constructed in a way to combine tissue from the same donor taken at different stages of the disease, that is, material collected from the primary tumor, a local recurrence, and a metastatic lesion, to allow for important longitudinal biological studies. Other TMAs combine different histological subtypes, for example, four well-known variants of adipocytic sarcomas on one array for comparative studies (e.g., UZL_PT_TMA_LPS) or one disease combining cases with diverse molecular drivers on one TMA (e.g., UZL_PT_TMA_GIST) (Table 2). The created panel of TMAs is linked with extensive, well-structured clinical information from the according donors.

### 3.3. Validation of TMAs

Once the TMA blocks were built, we cut at least 15 ready-to-use TMA sections and stored them in sealed containers. For long-term preservation and future experiments, some of precut TMAs were coated with paraffin [19]. Sections from variable TMAs have already been used successfully for a series of ongoing translational research projects, with a focus on the identification of novel biomarkers and screening for drug targets. A relatively big number of cases with STS could be easily tested in a single batch using immunostaining, and images could be automatically digitalized for data analysis and data sharing.

**Figure 1:**
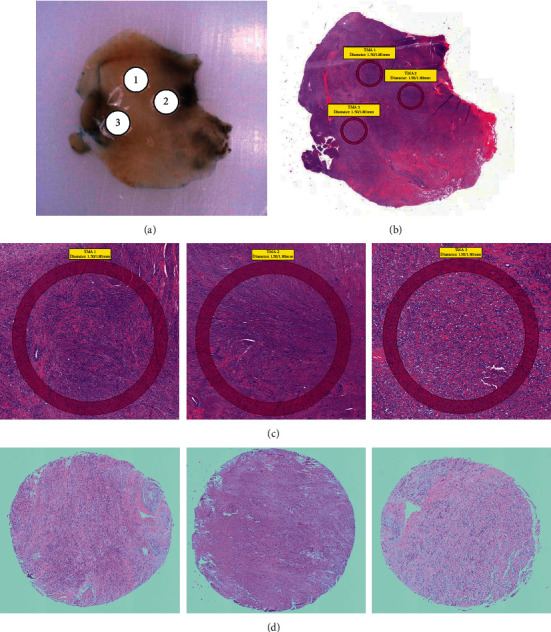
Example of triplicate tissue cores transferred from an inflammatory myofibroblastic tumor block to tissue microarray and the annotations on digital hematoxylin and eosin-stained slide images. Representative images of (a) an inflammatory myofibroblastic tumor block and (b) its whole tissue section stained with hematoxylin and eosin demonstrated the identical areas of interest used for the construction of respective TMA (CREATE_TMA_IMFT). (c) Microscopic images for annotations on an inflammatory myofibroblastic tumor whole tissue section stained with hematoxylin and eosin and (d) tissue cores of CREATE_TMA_IMFT stained with hematoxylin and eosin showed comparable histological characteristics.

To determine the tissue retrieval accuracy and histological consistency during the process of TMA construction, we compared the tissue morphology between donor block and TMA. Example images from an IMFT donor block and its H&E-stained section demonstrated great alignment between donor tissue and annotated areas of interest used for the construction of IMFT-related TMA (CREATE_TMA_IMFT) (Figures 1(a) and 1(b)). Triplicate annotations on H&E image showed the histology of (myo)fibroblastic spindle cells with inflammatory infiltration, which reflected on corresponding cores on CREATE_TMA_IMFT, and they also represented a morphological heterogeneity among cores (Figures 1(c) and 1(d)). To ensure the representativeness of antigenicity and molecular heterogeneity, we also compared the performance of IHC in whole tissue sections (original tumors) and TMAs constructed from STSs (Supplementary Figure S1). A high concordance of results was therefore determined (Supplementary Table S4). We concluded that the morphological and immunohistochemical characteristics of donor tissue and TMA tissue are well matched.

### 3.4. Exploration of TMAs for Target Expression

**Figure 2:**
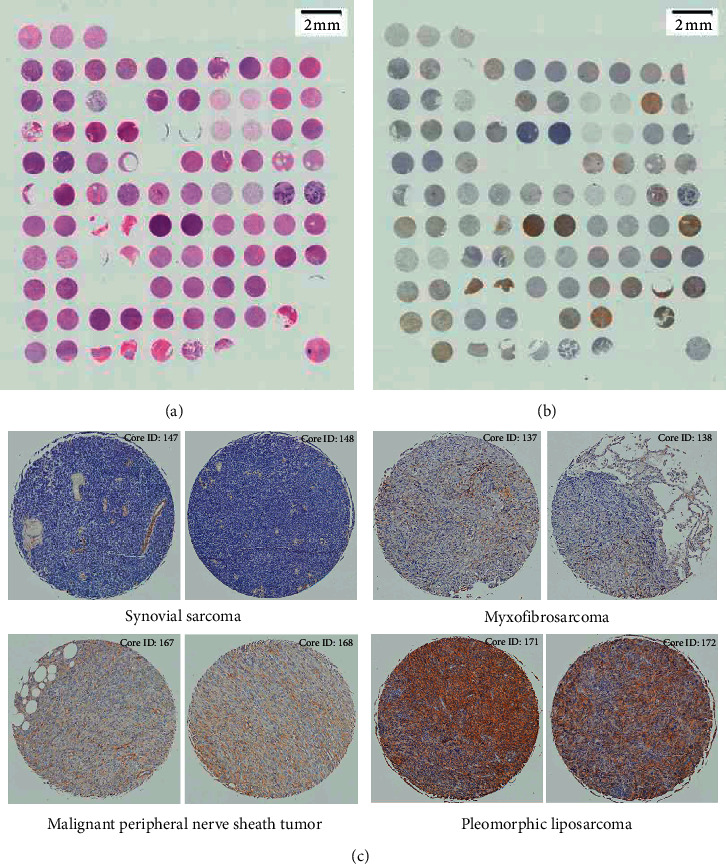
Example of hematoxylin and eosin and immunohistochemical staining on tissue microarray combining multiple subtypes of soft tissue sarcoma for rapid target screening purposes. An overview of tissue microarray sections stained with (a) hematoxylin and eosin and (b) immunohistochemical staining for PDGFR-B. (c) Images of duplicate cores from individual cases showing different levels of PDGFR-B expression in different subtypes of soft tissue sarcoma.

To test the usefulness of the platform, immunohistochemical and immunofluorescence assays were performed on TMA sections. As an illustration, a TMA containing various STS subtypes (UZL_PT_TMA_multiple subtype STS) was used to screen for PDGFR-B expression, a potential target for a drug that we later tested in the laboratory in mouse models of STS [20]. The TMA was successfully stained with H&E as well as with an anti-PDGFR-B antibody (Figures 2(a) and 2(b)), and each core was scored blindly by two independent researchers. Across 202 analyzed cores, 16.3% were scored as strongly positive, moderately positive (24.3%), weakly positive (30.2%), and negative (18.8%) and 10.4% was not evaluable. The results of staining per tumor type were summarized in Table 3. Examples of high-resolution scanned images showed negative, weakly positive, moderately positive, and strongly positive PDGFR-B expression in cases of SynSa, myxofibrosarcoma, MPNST, and PLPS, respectively (Figure 2(c)).

**Figure 3:**
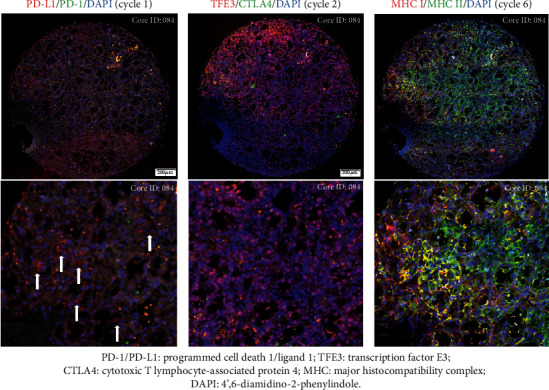
Example of marker expression using multiplex immunofluorescence (MILAN), on tissue microarray from tissue cores of alveolar soft part sarcoma. Representative fluorescence immunostainings of tissue cores and different cycles of the identical core showed the stability of tissue microarray throughout experiments. Images were digitally scanned under 200-fold magnification. The blue color showed DAPI staining and represented nucleated cells. Membrane expression of programmed cell death ligand 1 (red) was detected in a small proportion of tumor cells in the first cycle (left column) but no expression for programmed cell death 1 (green) was observed. In the middle column, pink color in merged images indicated nuclear localization of transcription factor E3 (red) but no green fluorescence for cytotoxic T lymphocyte-associated protein 4 was detected. In the sixth cycle (right column), membrane expression of major histocompatibility complex I/II and their coexpression were demonstrated in red, green, and yellow, respectively.

In the next step, we assessed the applicability of the multiplex immunofluorescence (MILAN) on TMAs. For this, we selected a TMA constructed from ASPS samples (CREATE_TMA_ASPS); ASPS is known in the clinic as one of the few STS subtypes with consistent response to treatment with immune checkpoint modifiers [21, 22]. We applied MILAN to the according TMA to investigate the complex immunological tumor microenvironment in this ultrarare entity. MILAN was successfully performed, and the presence of the immune checkpoint proteins, ASPS specific molecule (TFE3) and immunological markers was evaluated with an average evaluable rate of 93% (range 90–95% per target) in seven cycles of immunofluorescence staining on a single TMA section (Supplementary Table S5). Examples of the nuclear expression of TFE3 and membrane expression of programmed cell death ligand 1 and major histocompatibility complex I/II were demonstrated in Figure 3. We concluded that TMAs are very useful for comprehensive multiplex immunofluorescence assay, despite tissue loss during the experimental procedures (heat-treatment, incubation, wash, removal of cover glass and stripping, etc.).

## 4. Discussion

The rarity of STS makes it difficult to collect an adequate number of cases and tissue blocks for studying these diseases, especially in the case of ultrarare subtypes, for example, CCSA, ASPS, IMFT, or others which account for less than 1% of mesenchymal malignancies. It is important to have a sufficient tissue collection to be able to explore the biology of sarcoma entities and to identify potential biomarkers in order to advance research and ultimately patient care. TMA has potential advantages as compared to conventional archival sarcoma tissue in histopathological, immunohistochemical, and genetic studies. For these reasons, we decided to construct TMAs for our explorative studies but also to make them available for research collaborations. Our collection was mainly built from left-over material from patients diagnosed locally, but also supplemented tissue from patients enrolled in a prospective clinical trial performed in multiple European institutions [9–11]. In order to identify representative tissue to build such a platform, sarcoma tissue samples were retrieved from archives, and the annotations of area of interest were reviewed by reference sarcoma pathologists. The LECTOR database, established at Department of General Medical Oncology at UZ Leuven, provided important clinical information and helped selecting appropriate cases for creation of TMAs. During the last 5 years, we were able to collect 561 FFPE tumor blocks from 428 STS patients, which resulted in the creation of 11 STS TMAs (STS TMA platform).

Our TMA platform contains arrays of diverse STS subtypes including some ultrarare entities, and the morphological and immunohistochemical characteristics of the tissue on the TMA are reflecting the histological and molecular characteristics of the original donor tumor blocks. In the past, other scientific groups have created different types of TMAs for sarcoma research. Lahat et al. described TMAs from STS with complex-karyotype (*n* = 205) to be used for the correlative studies [23]. An LMS-specific TMA (*n* = 47) was described in the literature to assess intra- and intertumor heterogeneity [24]. TMAs from two prospective sarcoma trials exploring preoperative treatments were constructed to correlate the expression of biomarkers with patient outcome [25]. Nielsen et al. performed histological analysis to distinguish SynSa from other subtypes using TMAs constructed from SynSa (*n* = 46), Ewing sarcoma (*n* = 5), SFT (*n* = 5), MPNST (*n* = 5), LMS (*n* = 5), myxoid fibrosarcoma (*n* = 5), hemangiopericytoma (*n* = 4), and uncertain subtypes (*n* = 7) [26]. These and other TMAs were used for correlative and molecular analysis which have shown their utilities in sarcoma research [27–30]. To our knowledge, the TMA platform that we have built over the past 5 years likely represents the biggest and most diverse collection of well-annotated TMAs from sarcoma patients. The spectrum of entities ranges from common subtypes such as LMS and LPS to ultrarare variants such as CCSA, ASPS, or IMFT. Although this is already a broad and very unique collection, we are still expanding the platform to include other relevant subtypes of STS. A TMA from our collection of SFT is currently being constructed in collaboration with University Hospital Erlangen, Erlangen (Germany), while the creation of TMAs from angiosarcoma and SynSa patients is in an advanced preparatory phase.

A key advantage of our STS TMA platform is the applicability of tissue-based techniques (e.g., IHC, FISH, and immunofluorescence) on multiple sarcoma samples at the same time, applying uniform experimental conditions. This allows us to rapidly evaluate molecules of interest in a large number of STS cases, saving precious tissue specimen. IHC is the easiest and the most commonly used approach to evaluate protein expression, as exemplified by our PDGFR-B staining. As all tumor samples are placed on a single TMA, the staining of all specimens can be performed at the same time under identical conditions, achieving highest experimental standards and saving assay volume. Moreover, recent advances in multiplex immunostaining techniques such as MILAN enable us to test multiple molecules on a single TMA slide [12], revealing the cellular composition, localization, and interactions between cells, as well as increasing the utility of very precious biological material.

Well-annotated TMA can be of a great value to study predictive and prognostic biomarkers and to evaluate potential treatment targets. For instance, TMA comprised (patient-paired) samples from progressive stages of disease was used to demonstrate the increased PD-L1 expression in a small proportion of metastatic or recurrent samples in various STS subtypes, indicating the potential use of immune checkpoint inhibitors in the metastatic setting [31]. A complex-karyotype STS study, showing matrix metalloproteinase 2 and p53 as potential prognostic biomarkers, proved the utility of TMA linked with clinical data for correlative analysis [23]. Our well-annotated TMA collection can be used to determine the prevalence of a given alteration in different sarcoma subtypes that can be used for screening of potentially actionable drug targets. In our collection, some subtype-specific TMAs contain primary tumor, local recurrence, and/or metastatic lesion collected from the same patient and therefore can be used to study the molecular evolution of such diseases. Even more, in the vast majority of cases, we have theoretical access to the original blocks for confirmatory studies.

Beyond the advantages of TMA, there are limitations or issues that may occur which can affect the performance of such assays. The most commonly raised controversy is the representativeness of tumor heterogeneity in a small volume of tissue in TMA. This problem can be solved by multiple cores (2–3 per specimen) from different tumor regions and a larger size of punches (1.0 or 1.5 mm diameter) [32], and this is how we constructed our TMAs. As a result, diverse expression of analyzed molecules can be observed in different cores from a single case, representing the heterogeneity observed in the tumor tissue section, as illustrated by our pMAPK and PDGFR-B staining. Another issue is the risk of losing tissue cores during TMA sectioning or experiments. The way to section a TMA may cause uninterpretable cores spanning the entire block, which can be avoided by sectioning along the whole width of the array [33]. Sectioning along the length may exert excessive shearing forces on the wax and increase the risk of fracture. On the other hand, during IHC procedure for instance, a tissue core may detach as a result of washing, heating, and the pretreatment steps.

In order to ensure the quality of the TMAs, the construction of TMA blocks is supported by established procedures for selection to production, which is referred by others as “next-generation TMA protocol” [34]. There are a number of factors that can affect the quality of TMAs. First of all, the quality of a donor block is determined by its availability and durability. The availability can be measured by at least 3 or 4 mm height of a block, which ensures that the tissue volume is enough for the TMA construction. The durability of a donor block depends on the length of time and the environment (humidity and temperature) of the block storage conditions. An old block may lose the antigenicity and become fragile over time, which should be excluded if it is dehydrated [35]. Secondly, the selection of donor tissue punching should reflect the morphological features of corresponding STS subtypes. Therefore, for our TMA, all core annotations were reviewed by reference sarcoma pathologists. Next, variation can occur in localizing needle to punch tissue out based on the annotations on the H&E slides, and the shape of donor tissue may slightly deform during sections or heating/cooling of FFPE blocks [36]. To improve the precision of tissue retrieval, digital annotations and fully automated tissue microarrayer were applied for a better alignment between annotated images and donor blocks. As for tissue transfer, various hollow cylinders with different diameters (0.6–2.0 mm) can be applied. Even though the bigger core size, the better intratumor heterogeneity of intratumors, the use of 2.0 mm cylinders increases the risk of donor blocks damage, especially in case of biopsy samples. To limit this risk while maintaining the specimen heterogeneity, to have better attachment of tissue and to make cutting easier, we used 1.0 or 1.5 mm cylinders for our TMA construction. Lastly, the risk of samples cross-contamination in the process of construction, potentially caused by the usage of the same punching device, has been raised as a serious limitation of the TMA construction. Vassella et al. assessed the contamination transferred between samples using pyrosequencing and showed no cross-contamination between different tissue samples punched with the same device [37].

In addition to the TMA construction, we have also explored the use of digital pathology in the image acquisition and analysis of TMAs. So far, we have established an experimental pipeline involving TMA staining, automatic image acquisition, data storage, and sharing, based on the Olympus BX43 microscopic system. Nevertheless, the advanced technologies of deep learning or artificial intelligence, automatically quantitative IHC, and immunofluorescence became available and have been extensively used in research laboratories. There are a number of commercial algorithms available and applicable, such as cellSens imaging software [38] and many others [39–41]. Open-source software such as ImageJ (National Institutes of Health, Bethesda, US) [42] can also be used to develop methods for evaluation. With the recent advances in computing and image analysis algorithms, we aim to integrate computer-aided image analysis into the existing pipeline to further expand the applicability and flexibility of our platform in the future.

We plan to use our sarcoma-oriented TMA platform to study the biology of various common or ultrarare sarcoma subtypes, to screen for potential actionable targets, and to select cases in preparation of further translational or clinical research. Some of our established TMAs have been already used for target identification and informed subsequent projects focusing on the development of innovative treatments, including cytotoxic prodrugs and tyrosine kinase inhibitors. For instance, the TMA from multiple subtypes of STS has been used to screen for a novel a receptor tyrosine kinase as a potential actionable target. We performed IHC to determine the overexpression of this receptor in certain subtypes of STS and selected relevant cases for further *in vivo* drug test of a novel tyrosine kinase inhibitor in sarcoma patient-derived xenograft mouse models, comparing high versus low expressing xenografts based on TMA findings. Our platform is readily available for collaborative studies with research partners who are interested using TMA for sarcoma-related research purposes.

## 5. Conclusion

Over a period of 5 years, we have been able to create a very comprehensive and well-annotated TMA platform for sarcoma research, covering considerable numbers of the most common and some ultrarare STS entities. First exploratory studies have demonstrated that these arrays lead to comparable results as conventional analysis utilizing single block and that the TMAs are very suitable for a broad range of morphological, immunohistochemical, and immunological analytical techniques. We are intending to use these and future TMAs for tumor biology studies, for screening of potentially actionable molecules or genetic targets in sarcomas, and for case selection in further experimental work. We are making this unique resource available for scientific projects in collaboration with research partners.

## Figures and Tables

**Table 1:** List of the established, ready-to-use tissue microarrays.

Institutions that contributed samples	Name of tissue microarrays	Soft tissue sarcoma subtypes included	Number of cases	Number of blocks	Number of cores per block	Core size (mm)
UZ Leuven and collaborating institutions	UZL_PT_TMA_multiple subtype STS	Angiosarcoma	7	7	2	1.0
		Dedifferentiated liposarcoma	10	10		
		Pleomorphic liposarcoma	10	10		
		Myxoid liposarcoma	10	10		
		Leiomyosarcoma	9	9		
		Malignant peripheral nerve sheath tumor	10	10		
		Myxofibrosarcoma	11	11		
		Rhabdomyosarcoma	7	7		
		Synovial sarcoma	10	10		
		Undifferentiated pleomorphic sarcoma	9	9		
	UZL_PT_TMA_CCSA	Clear cell sarcoma	22	35	2	1.0
	UZL_PT_TMA_ASPS	Alveolar soft part sarcoma	12	16	3	1.5
	UZL_PT_TMA_IMFT	Inflammatory myofibroblastic tumor	12	12	3	1.5
	UZL_PT_TMA_ARMS	Alveolar rhabdomyosarcoma	21	34	2	1.0
	UZL_PT_TMA_LMS	Leiomyosarcoma	55	131	2	1.0
	UZL_PT_TMA_GIST	Gastrointestinal stromal tumor	72	76	2	1.0
	UZL_PT_TMA_LPS	Well-differentiated liposarcoma	14	20	3	1.0
		Dedifferentiated liposarcoma	11	16		
		Pleomorphic liposarcoma	6	7		
		Myxoid liposarcoma	10	19		
	UZL_PT_TMA_SFT	Solitary fibrous tumor	Under construction			
	UZL_PT_TMA_ANGS	Angiosarcoma	In preparation			
	UZL_PT_TMA_SYNSA	Synovial sarcoma	In preparation			

EORTC phase 2 trial 90101 (CREATE)	CREATE_TMA_CCSA	Clear cell sarcoma	32	32	3	1.5
	CREATE_TMA_ASPS	Alveolar soft part sarcoma	47	49	3	1.5
	CREATE_TMA_IMFT	Inflammatory myofibroblastic tumor	21	21	3	1.5

EORTC: European Organisation for Research and Treatment of Cancer

**Table 2:** Special subtype-specific tissue microarrays for (longitudinal) tumor biology studies.

Name of tissue microarrays	Soft tissue sarcoma subtypes	Different stages of samples in pairs	Number of cases
UZL_TMA_CCSA	Clear cell sarcoma	Primary tumor, metastatic lesion, and/or local recurrence	5
UZL_TMA_ASPS	Alveolar soft part sarcoma	Primary tumor and metastatic lesions	2
UZL_TMA_ARMS	Alveolar rhabdomyosarcoma	Primary tumor, metastatic lesion, and/or local recurrence	4
UZL_TMA_LMS	Leiomyosarcoma	Primary tumor, metastatic lesion, and/or local recurrence	36

		Different types of mutations	
UZL_TMA_GIST	Gastrointestinal stromal tumor	*KIT* exon 9	9
		*KIT* exon 11 deletion including codons 557/558	10
		*KIT* exon 11 substitutions	9
		*KIT* exon 11 duplication	7
		*KIT* exon 11 deletion outside codons 557/558	5
		*KIT* exon 11 + 17	5
		*KIT* exon 11 + 13	4
		*KIT* exon 11 p. W557_K558 deletion on codon 557–558	4
		*PDGFRA p*. D842 V	7
		*PDGFRA* other exon 18 mutation	5
		No *KIT/PDGFRA* mutation	7
		Different subgroups of LPS	
UZL_TMA_LPS	Liposarcoma	Well differentiated	14
		Dedifferentiated	11
		Myxoid/round cell	10
		Pleomorphic	6

PDGFR-A: platelet-derived growth factor receptor-alpha.

**Table 3:** Summarized results of immunohistochemistry for platelet-derived growth factor receptor beta on tissue microarray combining multiple subtypes of soft tissue sarcoma.

Soft tissue sarcoma subtypes	Total number of cases	Unevaluable	Negative	Weakly positive	Moderately positive	Strongly positive
Angiosarcoma	7	0	1	2	4	0
Dedifferentiated liposarcoma	10	0	0	1	5	4
Leiomyosarcoma	9	1	1	2	3	2
Malignant peripheral nerve sheath tumor	10	0	1	5	2	2
Myxoid round cell liposarcoma	10	0	3	6	1	0
Myxofibrosarcoma	11	1	1	3	2	4
Pleomorphic liposarcoma	10	0	0	3	3	4
Rhabdomyosarcoma	7	1	2	1	2	1
Synovial sarcoma	10	0	5	2	3	0
Undifferentiated pleomorphic sarcoma	9	1	0	0	5	3

## Data Availability

The data used to support the findings of this study are included within this aritcle and the supplementary information file(s). Relevant clinical data and additional images are available upon reasonable request from the corresponding author.

## Supplementary Materials

Supplementary Materials Supplementary Table S1: detailed information of antibody panel for multiplex immunostaining assay (MILAN) used to characterize immunological components in alveolar soft part sarcoma tissue microarray. Supplementary Table S2: characteristics of patients (*n* = 328) and donor tissue samples (*n* = 459) included in tissue microarrays constructed from specimens from University Hospitals Leuven, Leiden University Medical Center, and University Hospital Zürich. Supplementary Table S3: characteristics of patients (*n* = 100) and donor tissue samples (*n* = 102) from the European Organisation for Research and Treatment of Cancer 90101 phase II trial “CREATE.” Supplementary Table S4: comparison of immunohistochemical staining between whole tissue section and cores on tissue microarray from soft tissue sarcomas. Supplementary Table S5: evaluable rate of tissue cores on alveolar soft sarcoma tissue microarray in each cycle of multiplex immunostaining (MILAN). Supplementary Figure S1: examples of immunohistochemical staining for (a) pMAPK and (b) pAKT on whole tissue sections (original tumor) and corresponding tissue cores on tissue microarray from soft tissue sarcomas.
