# Supplementary material for: Establishment of an Academic Tissue Microarray Platform as a Tool for Soft Tissue Sarcoma Research
Source: Sarcoma. 2021 Mar 15;2021:6675260. doi: 10.1155/2021/6675260 (PMC8369337; doi:10.1155/2021/6675260)
Supplement: Supplementary Materials — Supplementary Table S1: detailed information of antibody panel for multiplex immunostaining assay (MILAN) used to characterize immunological components in alveolar soft part sarcoma tissue microarray. Supplementary Table S2: characteristics of patients (n = 328) and donor tissue samples (n = 459) included in tissue microarrays constructed from specimens from University Hospitals Leuven, Leiden University Medical Center, and University Hospital Zürich. Supplementary Table S3: characteristics of patients (n = 100) and donor tissue samples (n = 102) from the European Organisation for Research and Treatment of Cancer 90101 phase II trial “CREATE.” Supplementary Table S4: comparison of immunohistochemical staining between whole tissue section and cores on tissue microarray from soft tissue sarcomas. Supplementary Table S5: evaluable rate of tissue cores on alveolar soft sarcoma tissue microarray in each cycle of multiplex immunostaining (MILAN). Supplementary Figure S1: examples of immunohistochemical staining for (a) pMAPK and (b) pAKT on whole tissue sections (original tumor) and corresponding tissue cores on tissue microarray from soft tissue sarcomas. [file 6675260.f1.zip › 6675260.f1/Supplementary Figure S1 (2).docx]

**
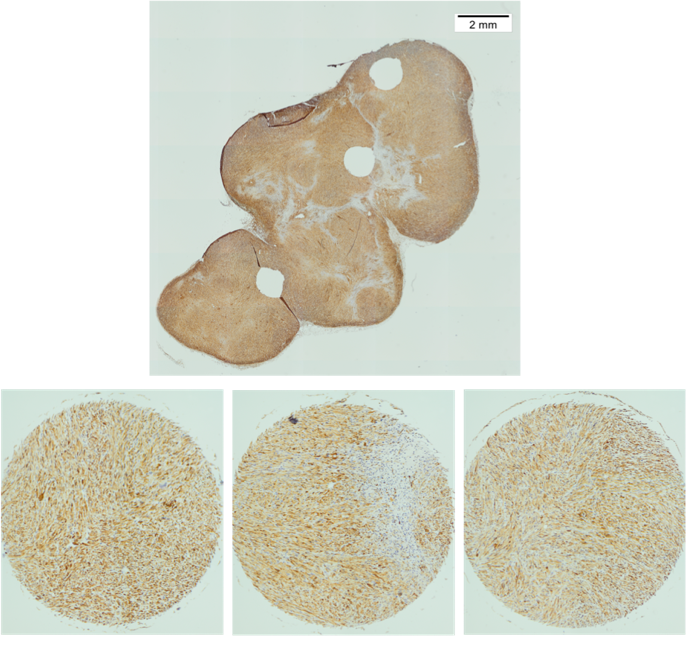

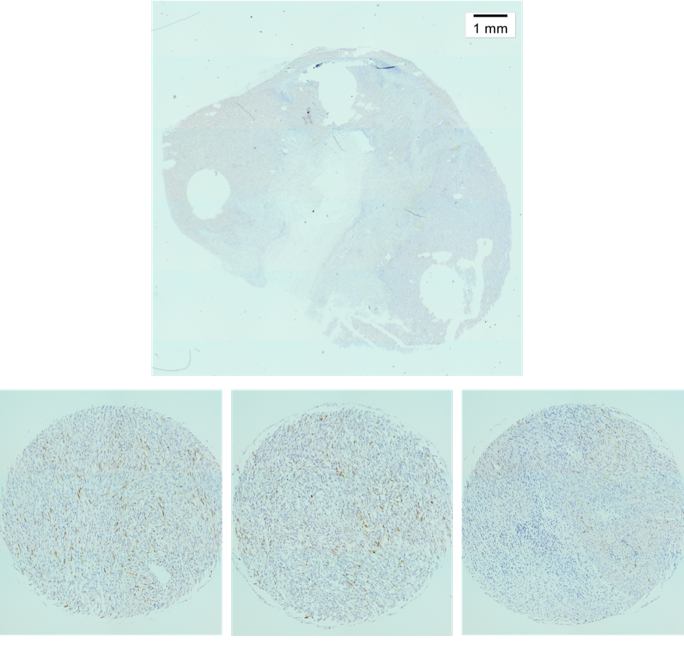
Supplementary Figure S1. Examples of immunohistochemical staining for (a) pMAPK and (b) pAKT on whole tissue sections (original tumors) and corresponding tissue cores on tissue microarray from soft tissue sarcomas**

(b)

(a)
