# Supplementary material for: Establishment of an Academic Tissue Microarray Platform as a Tool for Soft Tissue Sarcoma Research
Source: Sarcoma. 2021 Mar 15;2021:6675260. doi: 10.1155/2021/6675260 (PMC8369337; doi:10.1155/2021/6675260)
Supplement: Supplementary Materials — Supplementary Table S1: detailed information of antibody panel for multiplex immunostaining assay (MILAN) used to characterize immunological components in alveolar soft part sarcoma tissue microarray. Supplementary Table S2: characteristics of patients (n = 328) and donor tissue samples (n = 459) included in tissue microarrays constructed from specimens from University Hospitals Leuven, Leiden University Medical Center, and University Hospital Zürich. Supplementary Table S3: characteristics of patients (n = 100) and donor tissue samples (n = 102) from the European Organisation for Research and Treatment of Cancer 90101 phase II trial “CREATE.” Supplementary Table S4: comparison of immunohistochemical staining between whole tissue section and cores on tissue microarray from soft tissue sarcomas. Supplementary Table S5: evaluable rate of tissue cores on alveolar soft sarcoma tissue microarray in each cycle of multiplex immunostaining (MILAN). Supplementary Figure S1: examples of immunohistochemical staining for (a) pMAPK and (b) pAKT on whole tissue sections (original tumor) and corresponding tissue cores on tissue microarray from soft tissue sarcomas. [file 6675260.f1.zip › 6675260.f1/Supplementary Table S1.docx]

## Supplementary Table S1. Detailed information of antibody panel for multiplex immunostaining assay (MILAN) used to characterize immunological components in alveolar soft part sarcoma tissue microarray

| **Cycle** | **Primary Ab** | **Clone** | **Host species** | **Company** | **Cat.** | **Corresponding fluorophore conjugated secondary Ab** | **Company** | **Cat.** |
| --- | --- | --- | --- | --- | --- | --- | --- | --- |
| 1 | Anti-PD1 | Nat105 | Mouse IgG1 | Abcam | ab52587 | Alexa Fluor® 488 Goat Anti-Mouse IgG, Fcγ subclass 1 specific | Jackson ImmunoResearch | 115-545-205 |
|  | Anti-PD-L1 | SP-142 | Rabbit | Abcam | ab228462 | Alexa Fluor® 594 Goat Anti-Rabbit IgG (H+L) | Jackson ImmunoResearch | 111-585-144 |
|  |  | EPR19759 | Rabbit | Abcam | ab213524 |  |  |  |
|  |  | 28-8 | Rabbit | Abcam | ab205921 |  |  |  |
| 2 | Anti-CTLA4 | 2F1 | Mouse IgG2a | Sigma Aldrich | SAB1403712 | Alexa Fluor® 488 Goat Anti-Mouse IgG, Fcγ subclass 2a specific | Jackson ImmunoResearch | 115-545-206 |
|  | Anti-TFE3 | HLHe33 | Rabbit | Sigma Aldrich | HPA023881 | Alexa Fluor® 594 Goat Anti-Rabbit IgG (H+L) | Jackson ImmunoResearch | 111-585-144 |
| 3 | Anti-CD68 | PG-M1 | Mouse IgG3 | ThermoFisher | MA5-12407 | Alexa Fluor® 488 Goat Anti-Mouse IgG, Fcγ subclass 3 specific | Jackson ImmunoResearch | 115-545-209 |
|  | Anti-CD14 | 5A3B11B5 | Mouse IgG2b | SCBT | sc-58951 | Alexa Fluor® 594 Goat Anti-Mouse IgG, Fcγ subclass 2b specific | Jackson ImmunoResearch | 115-585-207 |
| 4 | Anti-NCAM | 123C3 | Mouse IgG1 | SCBT | sc-7326 | Alexa Fluor® 488 Goat Anti-Mouse IgG, Fcγ subclass 1 specific | Jackson ImmunoResearch | 115-545-205 |
|  | Anti-CD3 | SP7 | Rabbit | ThermoFisher | MA1-90582 | Alexa Fluor® 594 Goat Anti-Rabbit IgG (H+L) | Jackson ImmunoResearch | 111-585-144 |
| 5 | Anti-CD8 | C8/144B | Mouse IgG1 | SCBT | sc-53212 | Alexa Fluor® 488 Goat Anti-Mouse IgG, Fcγ subclass 1 specific | Jackson ImmunoResearch | 115-545-205 |
|  | Anti-CD4 | EPR6855 | Rabbit | Abcam | ab133616 | Alexa Fluor® 594 Goat Anti-Rabbit IgG (H+L) | Jackson ImmunoResearch | 111-585-144 |
| 6 | Anti-HLA Class 1 ABC | EMR8-5 | Mouse IgG1 | Abcam | ab70328 | Alexa Fluor® 488 Goat Anti-Mouse IgG, Fcγ subclass 1 specific | Jackson ImmunoResearch | 115-545-205 |
|  | Anti-HLA-DR | SPM289 | Mouse IgG2b | SCBT | sc-56545 | Alexa Fluor® 594 Goat Anti-Mouse IgG, Fcγ subclass 2b specific | Jackson ImmunoResearch | 115-585-207 |
| 7 | Anto-FOXP3 | 236A/E7 | Mouse IgG1 | Abcam | ab20034 | Alexa Fluor® 488 Goat Anti-Mouse IgG, Fcγ subclass 1 specific | Jackson ImmunoResearch | 115-545-205 |

PD-1/L1: program death 1/ligand 1, CTLA-4: cytotoxic T-lymphocyte-associated protein 4, TFE3: transcription factor E3, NCAM: Neural cell adhesion molecule, HLA: human leukocyte antigen, FOXP3: forkhead box P3
