# Supplementary material for: Establishment of an Academic Tissue Microarray Platform as a Tool for Soft Tissue Sarcoma Research
Source: Sarcoma. 2021 Mar 15;2021:6675260. doi: 10.1155/2021/6675260 (PMC8369337; doi:10.1155/2021/6675260)
Supplement: Supplementary Materials — Supplementary Table S1: detailed information of antibody panel for multiplex immunostaining assay (MILAN) used to characterize immunological components in alveolar soft part sarcoma tissue microarray. Supplementary Table S2: characteristics of patients (n = 328) and donor tissue samples (n = 459) included in tissue microarrays constructed from specimens from University Hospitals Leuven, Leiden University Medical Center, and University Hospital Zürich. Supplementary Table S3: characteristics of patients (n = 100) and donor tissue samples (n = 102) from the European Organisation for Research and Treatment of Cancer 90101 phase II trial “CREATE.” Supplementary Table S4: comparison of immunohistochemical staining between whole tissue section and cores on tissue microarray from soft tissue sarcomas. Supplementary Table S5: evaluable rate of tissue cores on alveolar soft sarcoma tissue microarray in each cycle of multiplex immunostaining (MILAN). Supplementary Figure S1: examples of immunohistochemical staining for (a) pMAPK and (b) pAKT on whole tissue sections (original tumor) and corresponding tissue cores on tissue microarray from soft tissue sarcomas. [file 6675260.f1.zip › 6675260.f1/Supplementary Table S2.docx]

| STS subtype | CCSA | ASPS | IMFT | ARMS | LMS | Myxoid /round cell LPS | PLPS | WDLPS | DDLPS | GIST | Angio-sarcoma | MPNST | Myxofibro-sarcoma | RMS | SynSa | UPS |
| --- | --- | --- | --- | --- | --- | --- | --- | --- | --- | --- | --- | --- | --- | --- | --- | --- |
|  |  |  |  |  |  |  |  |  |  |  |  |  |  |  |  |  |
| Total number of cases | 22 | 12 | 12 | 21 | 64 | 19 | 16 | 14 | 22 | 72 | 7 | 10 | 11 | 7 | 10 | 9 |
| **Gender** |  |  |  |  |  |  |  |  |  |  |  |  |  |  |  |  |
| Male | 13 (59%) | 3 (25%) | 4 (33%) | 11 (52%) | 25 (39%) | 10 (53%) | 9 (56%) | 8 (57%) | 12 (55%) | 36 (50%) | 2 (29%) | 6 (60%) | 5 (45%) | 6 (86%) | 6 (60%) | 1 (11%) |
| Female | 9 (41%) | 5 (42%) | 3 (25%) | 10 (48%) | 39 (61%) | 9 (47%) | 7 (44%) | 6 (43%) | 10 (45%) | 36 (50%) | 5 (71%) | 4 (40%) | 6 (55%) | 1 (14%) | 4 (40%) | 8 (89%) |
| Unknown | 0 | 4 (33%) | 5 (42%) | 0 | 0 | 0 | 0 | 0 | 0 | 0 | 0 | 0 | 0 | 0 | 0 | 0 |
| **Age at diagnosis**  **(years)** |  |  |  |  |  |  |  |  |  |  |  |  |  |  |  |  |
| <20 | 1 (5%) | 3 (25%) | 1 (8%) | 11 (52%) | 0 | 1 (5%) | 0 | 0 | 0 | 0 | 0 | 1 (10%) | 0 | 1 (14%) | 0 | 0 |
| 20-39 | 9 (41%) | 3 (25%) | 1 (8%) | 6 (29%) | 3 (5%) | 6 (32%) | 0 | 1 (7%) | 2 (9%) | 6 (8%) | 0 | 7 (70%) | 0 | 2 (29%) | 2 (20%) | 0 |
| 40-60 | 9 (41%) | 1 (8%) | 4 (33%) | 4 (19%) | 28 (44%) | 9 (47%) | 7 (44%) | 7 (50%) | 6 (27%) | 32 (44%) | 4 (57%) | 0 | 5 (45%) | 0 | 5 (50%) | 0 |
| >60 | 3 (14%) | 1 (8%) | 1 (8%) | 0 | 32 (50%) | 3 (16%) | 9 (56%) | 6 (43%) | 14 (64%) | 34 (47%) | 3 (43%) | 2 (20%) | 6 (55%) | 4 (57%) | 3 (30%) | 9 (100%) |
| Unknown | 0 | 4 (33%) | 5 (42%) | 0 | 1 (2%) | 0 | 0 | 0 | 0 | 0 | 0 | 0 | 0 | 0 | 0 | 0 |
|  |  |  |  |  |  |  |  |  |  |  |  |  |  |  |  |  |
| Total number of tumor samples | 35 | 16 | 12 | 34 | 140 | 28 | 17 | 20 | 27 | 76 | 7 | 10 | 11 | 7 | 10 | 9 |
| **Origin of lesion** |  |  |  |  |  |  |  |  |  |  |  |  |  |  |  |  |
| Primary tumor | 20 (57%) | 7 (44%) | 6 (50%) | 11 (32%) | 43 (31%) | 10 (36%) | 14 (82%) | 15 (75%) | 9 (33%) | 46 (61%) | 0 | 2 (20%) | 0 | 3 (43%) | 0 | 3 (33%) |
| Local relapse | 2 (6%) | 0 | 0 | 3 (9%) | 14 (10%) | 4 (14%) | 1 (6%) | 5 (25%) | 12 (44%) | 25 (33%) | 1 (14%) | 1 (10%) | 1 (9%) | 0 | 0 | 4 (44%) |
| Metastatic lesion | 13 (37%) | 5 (31%) | 1 (8%) | 20 (59%) | 83 (59%) | 11 (39%) | 2 (12%) | 0 | 6 (22%) | 5 (7%) | 6 (86%) | 7 (70%) | 10 (91%) | 4 (57%) | 10 (100%) | 2 (22%) |
| Unknown | 0 | 4 (25%) | 5 (42%) | 0 | 0 | 3 (11%) | 0 | 0 | 0 | 0 | 0 | 0 | 0 | 0 | 0 | 0 |
| **Anatomical location** |  |  |  |  |  |  |  |  |  |  |  |  |  |  |  |  |
| Abdomen and thorax | 9 (26%) | 3 (19%) | 3 (25%) | 5 (15%) | 97 (69%) | 7 (25%) | 5 (29%) | 14 (70%) | 20 (74%) | 74 (87%) | 5 (71%) | 7 (70%) | 5 (45%) | 3 (43%) | 8 (80%) | 2 (22%) |
| Extremities | 14 (40%) | 5 (31%) | 3 (25%) | 9 (26%) | 26 (19%) | 13 (46%) | 10 (59%) | 2 (10%) | 7 (26%) | 0 | 1 (14%) | 1 (10%) | 2 (18%) | 2 (29%) | 0 | 6 (67%) |
| Head and neck region | 2 (6%) | 2 (13%) | 1 (8%) | 5 (15%) | 8 (6%) | 3 (11%) | 0 | 3 (15%) | 0 | 1 (1%) | 1 (14%) | 0 | 0 | 0 | 0 | 0 |
| Trunk | 1 (3%) | 2 (13%) | 0 | 15 (44%) | 9 (6%) | 1 (4%) | 1 (6%) | 1 (5%) | 0 | 1 (1%) | 0 | 0 | 0 | 1 (14%) | 0 | 0 |
| Unknown | 9 (26%) | 4 (25%) | 5 (42%) | 0 | 0 | 4 (14%) | 1 (6%) | 0 | 0 | 0 | 0 | 2 (20%) | 4 (36%) | 1 (14%) | 2 (20%) | 1 (11%) |

**Supplementary Table S2. Characteristics of patients (n=328) and donor tissue samples (n=459) included in tissue microarrays constructed from specimens from University Hospitals Leuven, Leiden University Medical Center and University Hospital Zürich**

STS: soft tissue sarcoma, CCSA: clear cell sarcoma, ASPS: alveolar soft part sarcoma, IMFT: inflammatory myofibroblastic tumor, ARMS: alveolar rhabdomyosarcoma, LMS: leiomyosarcoma, LPS: liposarcoma, PLPS: pleomorphic liposarcoma, WDLPS: well-differentiated liposarcoma, DDLPS: dedifferentiated liposarcoma, GIST: gastrointestinal stromal tumor, MPNST: malignant peripheral nerve sheath tumor, RMS: rhabdomyosarcoma, SynSa: synovial sarcoma, UPS: undifferentiated pleomorphic sarcoma
