# Supplementary material for: Establishment of an Academic Tissue Microarray Platform as a Tool for Soft Tissue Sarcoma Research
Source: Sarcoma. 2021 Mar 15;2021:6675260. doi: 10.1155/2021/6675260 (PMC8369337; doi:10.1155/2021/6675260)
Supplement: Supplementary Materials — Supplementary Table S1: detailed information of antibody panel for multiplex immunostaining assay (MILAN) used to characterize immunological components in alveolar soft part sarcoma tissue microarray. Supplementary Table S2: characteristics of patients (n = 328) and donor tissue samples (n = 459) included in tissue microarrays constructed from specimens from University Hospitals Leuven, Leiden University Medical Center, and University Hospital Zürich. Supplementary Table S3: characteristics of patients (n = 100) and donor tissue samples (n = 102) from the European Organisation for Research and Treatment of Cancer 90101 phase II trial “CREATE.” Supplementary Table S4: comparison of immunohistochemical staining between whole tissue section and cores on tissue microarray from soft tissue sarcomas. Supplementary Table S5: evaluable rate of tissue cores on alveolar soft sarcoma tissue microarray in each cycle of multiplex immunostaining (MILAN). Supplementary Figure S1: examples of immunohistochemical staining for (a) pMAPK and (b) pAKT on whole tissue sections (original tumor) and corresponding tissue cores on tissue microarray from soft tissue sarcomas. [file 6675260.f1.zip › 6675260.f1/Supplementary Table S3 .docx]

| **STS subtype** | **CCSA** | **ASPS** | **IMFT** |
| --- | --- | --- | --- |
| Total number of cases | 32 | 47 | 21 |
| **Gender** |  |  |  |
| Male | 18 (56%) | 25 (53%) | 10 (48%) |
| Female | 12 (38%) | 21 (45%) | 8 (38%) |
| Unknown | 2 (6%) | 1 (2%) | 3 (14%) |
| **Age at diagnosis (years)** |  |  |  |
| <20 | 1 (3%) | 7 (15%) | 1 (5%) |
| 20-39 | 12 (38%) | 32 (68%) | 5 (24%) |
| 40-60 | 16 (50%) | 6 (13%) | 6 (29%) |
| >60 | 1 (3%) | 1 (2%) | 6 (29%) |
| Unknown | 2 (6%) | 1 (2%) | 3 (14%) |
|  |  |  |  |
| Total number of tumor samples | 32 | 49 | 21 |
| **Origin of selected sample** |  |  |  |
| Primary tumor | 20 (63%) | 37 (76%) | 15 (71%) |
| Metastatic lesion | 11 (34%) | 11 (22%) | 6 (29%) |
| Unknown | 1 (3%) | 1 (2%) | 0 |
|  |  |  |  |
| **Molecular alteration status (by FISH)** | ***EWSR1*** | ***TFE3*** | ***ALK*** |
| Detected | 28 (88%) | 44 (90%) | 13 (62%) |
| Not detected | 3 (9%) | 4 (8%) | 8 (38%) |
| Unknown | 1 (3%) | 1 (2%) | 0 |

**Supplementary Table S3. Characteristics of patients (n=100) and donor tissue samples (n=102) from the European Organisation for Research and Treatment of Cancer 90101 phase II trial "CREATE"**

STS: soft tissue sarcoma, CCSA: clear cell sarcoma, ASPS: alveolar soft part sarcoma, IMFT: inflammatory myofibroblastic tumor, EWSR1: Ewing sarcoma breakpoint region 1, TFE3: transcription factor E3, ALK: anaplastic lymphoma kinase
