# Supplementary material for: Establishment of an Academic Tissue Microarray Platform as a Tool for Soft Tissue Sarcoma Research
Source: Sarcoma. 2021 Mar 15;2021:6675260. doi: 10.1155/2021/6675260 (PMC8369337; doi:10.1155/2021/6675260)
Supplement: Supplementary Materials — Supplementary Table S1: detailed information of antibody panel for multiplex immunostaining assay (MILAN) used to characterize immunological components in alveolar soft part sarcoma tissue microarray. Supplementary Table S2: characteristics of patients (n = 328) and donor tissue samples (n = 459) included in tissue microarrays constructed from specimens from University Hospitals Leuven, Leiden University Medical Center, and University Hospital Zürich. Supplementary Table S3: characteristics of patients (n = 100) and donor tissue samples (n = 102) from the European Organisation for Research and Treatment of Cancer 90101 phase II trial “CREATE.” Supplementary Table S4: comparison of immunohistochemical staining between whole tissue section and cores on tissue microarray from soft tissue sarcomas. Supplementary Table S5: evaluable rate of tissue cores on alveolar soft sarcoma tissue microarray in each cycle of multiplex immunostaining (MILAN). Supplementary Figure S1: examples of immunohistochemical staining for (a) pMAPK and (b) pAKT on whole tissue sections (original tumor) and corresponding tissue cores on tissue microarray from soft tissue sarcomas. [file 6675260.f1.zip › 6675260.f1/Supplementary Table S4 (2).docx]

**Supplementary Table S4. Comparison of immunohistochemical staining between whole tissue section (original tumors) and cores on tissue microarray from soft tissue sarcomas**

| **Soft tissue sarcoma subtypes** | **Name of cases** | **pMAPK expression score** | | **pAKT expression score** | |
| --- | --- | --- | --- | --- | --- |
|  |  | **Whole tissue section** | **Tissue microarray*** | **Whole tissue section** | **Tissue microarray*** |
| Dedifferentiated liposarcoma | DDLPS_1 | 2 | 2 | 0 | 0 |
|  | DDLPS_2 | 3 | 3 | 0 | 0 |
|  | DDLPS_3 | 1 | 1 | 0 | 0 |
| Leiomyosarcoma | LMS_1 | 2 | 2 | 1 | 1 |
|  | LMS_2 | 2 | 2 | 1 | 1 |
|  | LMS_3 | 2 | 2 | 0 | 0 |
|  | LMS_4 | 2 | 2 | 0 | 0 |
| Myxofibrosarcoma | MFS_1 | 2 | 2 | 0 | 0 |
|  | MFS_2 | 1 | 1 | 1 | 1 |
|  | MFS_3 | 1 | 1 | 1 | 1 |
|  | MFS_4 | 1 | 1 | 1 | 1 |
|  | MFS_5 | 2 | 2 | 0 | 0 |
|  | MFS_6 | 2 | 1 | 0 | 0 |

0: negative, 1: weakly positive, 2: moderately positive, 3: strongly positive; *Tissue microarrays are presented as mean value from 3 cores; pMAPK: phosphorylation form of mitogen-activated protein kinase, pAKT: phosphorylation form of AKT
